# Expansion and Molecular Characterization of AP2/ERF Gene Family in Wheat (*Triticum aestivum* L.)

**DOI:** 10.3389/fgene.2021.632155

**Published:** 2021-03-31

**Authors:** Muhammad Waheed Riaz, Jie Lu, Liaqat Shah, Liu Yang, Can Chen, Xu Dong Mei, Liu Xue, Muhammad Aamir Manzoor, Muhammad Abdullah, Shamsur Rehman, Hongqi Si, Chuanxi Ma

**Affiliations:** ^1^College of Agronomy, Anhui Agricultural University, Hefei, China; ^2^Key Laboratory of Wheat Biology and Genetic Improvement on Southern Yellow & Huai River Valley, Ministry of Agriculture and Rural Affairs, Hefei, China; ^3^Department of Botany, Mir Chakar Khan Rind University, Sibi, Pakistan; ^4^School of Life Sciences, Anhui Agricultural University, Hefei, China; ^5^National Engineering Laboratory of Crop Stress Resistance Breeding, School of Life Sciences, Anhui Agricultural University, Hefei, China; ^6^National United Engineering Laboratory for Crop Stress Resistance Breeding, Hefei, China; ^7^Anhui Key Laboratory of Crop Biology, Hefei, China

**Keywords:** AP2/ERF, phylogenetic analysis, expansion, evolution, expression pattern, qRT-PCR

## Abstract

The AP2/ERF is a large protein family of transcription factors, playing an important role in signal transduction, plant growth, development, and response to various stresses. AP2/ERF super-family is identified and functionalized in a different plant but no comprehensive and systematic analysis in wheat (*Triticum aestivum* L.) has been reported. However, a genome-wide and functional analysis was performed and identified 322 TaAP2/ERF putative genes from the wheat genome. According to the phylogenetic and structural analysis, TaAP2/ERF genes were divided into 12 subfamilies (Ia, Ib, Ic, IIa, IIb, IIc, IIIa, IIIb, IIIc, IVa, IVb, and IVc). Furthermore, conserved motifs and introns/exons analysis revealed may lead to functional divergence within clades. *Cis*-Acting analysis indicated that many elements were involved in stress-related and plant development. Chromosomal location showed that 320 AP2/ERF genes were distributed among 21 chromosomes and 2 genes were present in a scaffold. Interspecies microsynteny analysis revealed that maximum orthologous between *Arabidopsis*, rice followed by wheat. Segment duplication events have contributed to the expansion of the AP2/ERF family and made this family larger than rice and *Arabidopsis*. Additionally, AP2/ERF genes were differentially expressed in wheat seedlings under the stress treatments of heat, salt, and drought, and expression profiles were verified by qRT-PCR. Remarkably, the RNA-seq data exposed that AP2/ERF gene family might play a vital role in stress-related. Taken together, our findings provided useful and helpful information to understand the molecular mechanism and evolution of the AP2/ERF gene family in wheat.

## Introduction

Plants have a typical and complicated regulation process to defend their growth, productivity, and development. These plant parts are suffering from the adverse effects of many abiotic stresses such as drought, high temperature and salinity. Plants have a response mechanism to thrive and grow under these environmental stresses by repressing or triggering the expression of numbers of the genes with specific functions (Sakuma et al., 2002; Wessler, 2005). Transcription factors, the regulators of gene expressions, play a key role in the signal transformation network. They directly turn on or off to target gene expression, thereby interrupting the interaction between different gene signaling pathways (Xu et al., 2008; Feng et al., 2015; Qin et al., 2017).

APETALA2/Ethylene Responsive Factor (AP2/ERF) gene family is the most extensive group of plant transcription factors (TFs) having a significant role in signal transduction, plant growth, and development, and also participating in biotic/abiotic stress response (Licausi et al., 2013). The AP2/ERF supergene family is characterized by a 57–66 amino acids conserved AP2 DNA binding domain (Okamuro et al., 1997). Generally, according to the sequence similarity and number of AP2 domains, the AP2/ERF gene family is further divided into five main groups: AP2, DREB, ERF, RAV, and Soloist group. AP2 TFs have two AP2 domains or one AP2 domain which are alike to the AP2 domain in the double domain group, but ERF group members have one AP2 domain (Nakano et al., 2006). The RAV group transcription factors only contain one AP2/ERF domain, but there is also an extra B3 domain in the RAV group. DREB and ERF groups have a single AP2/ERF domain. A small part of the transcription factor with a large deviation from an AP2 domain (similar to AP2 domain) and the gene structure is named as Soloist group (Sakuma et al., 2002).

It was previously thought that the supergene family of AP2/ERF only exists in plants, but current research showed that AP2/ERF transcription factors also exist in protists and ciliates (Rashid et al., 2012; Licausi et al., 2013). With the development of Science and Scientific knowledge of plant genome sequences, AP2/ERF gene family have been reported in several plants like *Arabidopsis*, Rice (Nakano et al., 2006), maize (Liu et al., 2013), Sorghum (Yan et al., 2013), Soybean (Zhang et al., 2008), and foxtail millet (Lata et al., 2014). The AP2 family in *Arabidopsis* is the most studied gene in this family. The number of AP2 genes identified in *Arabidopsis* was eighteen (Sakuma et al., 2002).

The gene family AP2/ERF play a crucial role in controlling plant growth and biotic and abiotic stresses tolerance (Moose and Sisco, 1996; Aukerman and Sakai, 2003; Jofuku et al., 2005; Lata and Prasad, 2011; Liu et al., 2013; Schmidt et al., 2013; Jiang et al., 2014; Zhu et al., 2014). Normally the AP2 Subfamily members were the key factors which are involved in regulating organ building and development, like leaf epidermal cell determinacy, floral organ patterning, and spikelet meristem differentiation (Aukerman and Sakai, 2003) as well as seed yield and seed mass (Jofuku et al., 2005; Taketa et al., 2008). The RAV subfamily is involved in the signal transduction of ethylene and other plant hormones (Alonso et al., 2003), Brassinosteroid (Hu et al., 2004), and also participated in the water stress tolerance of DREB subfamily members. The DREB genes with a conservative binding sequence CCGAC commonly bind to the main LTR and/or MBS (core sequences: CCGAAA and C/TAACTG) *cis*-acting elements upstream of the gene and plays a crucial role in resisting biological stress (including cold and drought) effect (Hogan et al., 2003; Wang et al., 2017). Additionally, the transcription factors from the ERF subfamily binds with GCC-boxes which are entangled in different pathways of hormone signaling, like ethylene, 2-hydroxybenzoic acid, and jasmonic acid pathways (Fujimoto et al., 2000; Oñate-Sánchez and Singh, 2002; Mantiri et al., 2008). The transcription factors from DREB subfamily which binds to the DRE/CRT elements are largely entangled in abiotic stresses like drought (Hong and Woo, 2005; Fang et al., 2015), freezing (Ito et al., 2006; Fang et al., 2015), heat (Qin et al., 2007), osmosis (Fujita et al., 2011), and salinity (Hong and Woo, 2005; Bouaziz et al., 2013).

The number of AP2 member gene reported in rice (*Oryza sativa* L.) was 23 (Rashid et al., 2012), among them various genes have been characterized functionally, including INDETERMINATE SPIKELET1 (OsIDS1) (Lee and An, 2012; Lee et al., 2014; Jiang et al., 2019), SMALL ORGAN SIZE1 (SMOS1) (Aya et al., 2014). OsIDS1 and SNB both possess a vital role in the establishment of floral meristems and inflorescence architecture (Lee and An, 2012). Newly, it was investigated that SNB controls seed size and seed shattering (Jiang et al., 2019). It was reported that AP2-like glossy15 in maize regulates the identity of leaf epidermal cells (Moose and Sisco, 1996).

In wheat, a TaERF1 gene may be affected by various environmental stresses including drought, salinity, cold and external hormones like ET, salicylic acid (SA), and ABA, also known as a pathogen (*Blumeria graminis* f. sp. tritici) resistance gene. Enhanced expression of TaERF1 in tobacco and *Arabidopsis* may enhance resistance to pathogens and increase tolerance to various abiotic stresses (Xu et al., 2007). Overexpression of *Haynaldia villosa* ERF1-V in wheat functions against powdery mildew, drought and salinity (Xing et al., 2017). Similarly, another gene in wheat belongs to ERF family, TaPIE1, which increases resistance to *Rhizoctonia cerealis* and activates the defense and stress-related signals. These signals act downstream of the ET signaling pathway, thereby increasing tolerance of wheat to freezing stress (Zhu et al., 2014).

These results of our finding help us to understand the role of AP2/ERF family genes in wheat, and give the foundation for further functional analysis of wheat AP2/ERF family genes.

## Materials and Methods

### Plant Materials and Stress Treatments

The seeds of hexaploid wheat (*Triticum aestivum* L. cv. Chinese Spring) were surface sterilized and grown in a petri dish with a small amount of water in a greenhouse for 2–3 days. At the appearance of cotyledon and plumule, the seeds were transferred to plastic pots containing soil and maintained in a growth chamber under a 16 h photoperiod (daytime temperature 22°C, night temperature 20°C) with a relative humidity of 60%. At the three-leaf stage of seedling development, these plants were exposed to 150 mM NaCl, 20% (w/v) Polyethylene glycol (PEG) 6000, and a high temperature of, 42°C. Seedling without this treatment served as controls. Post-treatment leaf tissues were collected at 0, 3, 12, and 24 h. All stressed and control leaf samples with three biological replicates at each point were collected and immediately frozen in liquid nitrogen. All the samples were then kept at −80°C until total RNA was extracted.

### Database Search and Identification and Characterization of AP2/ERF Genes in Wheat Genome

The sequencing of wheat genome was completed in 2018 (Appels et al., 2018), and coding and protein sequencing are also available^1^. All the protein sequencing containing AP2 domain were downloaded from Ensemble Plants database^2^. The AP2/ERF domain (PF00847) was retrieved from the PFAM database^3^ used as a query for Hidden Markov Model (HMM) search using HMMER 3.0 software with an E < 1e^–5^ threshold predefined. The sequences were further analyzed by SMART^4^ (Letunic et al., 2015) and the sequences were removed without AP2 domains by searching in the PFAM databases (see text footnote 3). To further confirm the presence of AP2 domain in the sequences, CDD^3^ was used (Marchler-Bauer et al., 2017). The sequences of theoretical isoelectric point (pI) and molecular weight were obtained by using the ExPASy tool online^5^. The gene ontology (GO) annotations and subcellular localization of all the TaAP2/ERF proteins were determined by using CELLO2GO tool (Yu et al., 2006; Faraji et al., 2020). The rice AP2 sequences were downloaded from Rice Genome Annotation Project (RGAP)^6^ (Kawahara et al., 2013) and *Arabidopsis* AP2 sequences from The *Arabidopsis* Information Resources (TIAR)^7^.

### Gene Structure, Multiple Sequence Alignment, and Phylogenetic Analysis

The structure of exon/intron was created by Gene Structure Display Server (GSDS)^8^ (Hu et al., 2015) by using coding sequences (CDS) and corresponding genomic sequences were downloaded from Ensemble plants website (see text footnote 2). ClustalW version 2.0 with default settings (Larkin et al., 2007) was used for multiple sequence alignments analysis. Phylogenetic tree and molecular evolutionary analysis was performed with Molecular Evolutionary Genetics Analysis (MEGA) version 6 software using the neighbor-joining (NJ) method with bootstrap analysis (1000 replicates) (Tamura et al., 2013).

### Gene Duplication Analysis

To find the duplicated gene pairs, we use the criteria set by Wang et al. (2016): (1) the nucleotide sequence which we were align covered >80% of the longer aligned gene, and the (2) identity between the aligned regions must be >80%.

### Motif Analysis

In order to identify the conserved motif of AP2/ERF protein sequences in wheat using Multiple Em for Motif Elicitation online web tool^9^. The parameters followed are: optimal motif width is 6–200 and the maximal number of motifs is 20.

### *Cis*-Acting Element Analysis and Microsynteny Analysis

For *cis*-acting element analysis 2000 bp upstream sequence from the initiation code (ATG) of genomic DNA sequence was downloaded. The presence of different *cis*-acting elements in the AP2/ERF gene family of wheat was determined by using PlantCARE software^10^ (Lescot et al., 2002). To obtain the microsynteny between *Triticum aestivum* L, *Oryza sativa* L, and *Arabidopsis thaliana*, Multiple Collinearity Scan toolkit (MCSscanX) was used.

### Analysis of Expression Pattern of TaAP2/ERF Genes

To check the tissue-specific expression of AP2/ERF genes in different wheat tissues (grain, leaf, root, spike, and stem) and under different abiotic stresses of 18 AP2/ERF genes, the data of SRP043554, SRP045409, and SRP041017 RNA-seq project from expVIP database^11^ (Zhang et al., 2014; Li et al., 2015; Liu et al., 2015; Borrill et al., 2016) and expression data of hexaploid bread wheat (var. Chinese Spring) obtained from WheatExp database^12^ (Pearce et al., 2015) were analyzed. Heatmaps were drawn, based on log 2 transcripts per million (TPM) and Fragments Per Kilobase of transcript per Million mapped reads (FPKM) values using TBtools.

### Extraction of RNA, cDNA Synthesis, and qRT-PCR Analysis

According to the manufacturer’s instructions, TransZOL (TransGEN Biotech, Beijing, China) was used to extract the Total RNA from the leaves of wheat seedlings. Used DeNovix DS-11 spectrophotometrically to check the quality and quantity of Isolated RNA’s and perform 2% agarose gel electrophoresis, and stored at −80°C for further use. According to the manufacturer’s instructions, we used HiScript^®^III RT SuperMix with + gDNA wiper (Vazyme, Nanjing, China) for cDNA synthesis. Quantitative real-time PCR (qRT-PCR) was performed using CFX96 Real-Time PCR Detection System (Bio-Rad, United States) and AceQ^®^ Universal SYBR^®^ qPCR Master Mix (Vazyme, Nanjing, China). The total reaction mixture for qRT-PCR was 20 μl including 10 μl 2 × AceQ Universal SYBR qPCR Master Mix, 2 μl cDNA, 1 μl forward primer (10 μmol), 1 μl reverse primer (10 μmol), and 6 μl RNAase free water. The parameters followed by qRT-PCR are 95°C for 5 min, and then 40 cycles of 95°C for 10 s and 60°C for 30 s. The wheat β*-actin* gene was used as an internal control for all qRT-PCR expression analysis (Hu et al., 2013). The 2*^–^*^Δ^^ΔCT^ method was applied to determine relative gene expression level (Livak and Schmittgen, 2001). In all our experiments we used three biological repeats and three technical repeats for all qRT-PCR analysis. All the primers used in qRT-PCR are listed in Supplementary Table 1.

## Results

### Data Searching, Identification, and Molecular Characterization of AP2/ERF Gene Family in Wheat

**FIGURE 1:**
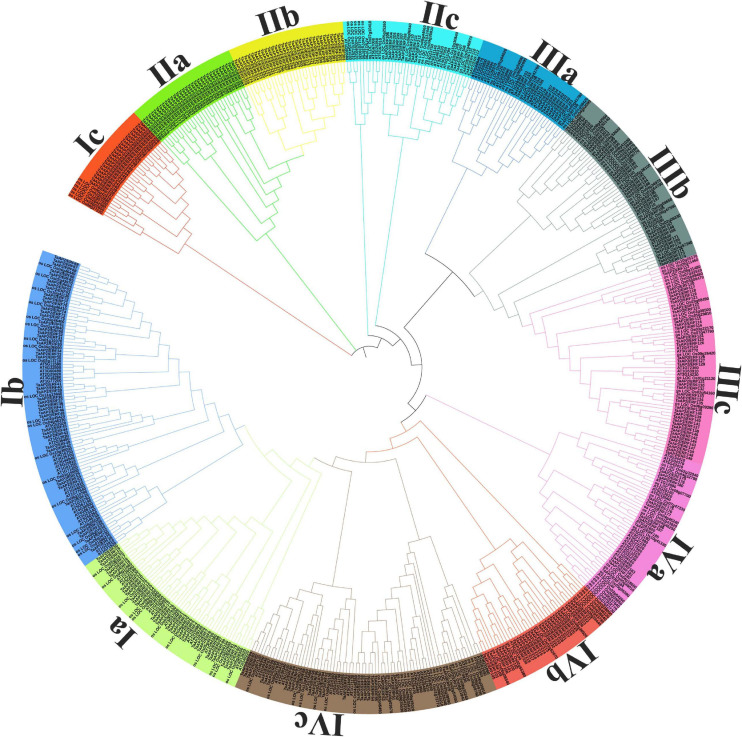
Phylogenetic tree of AP2/ERF genes in Wheat, Rice, and *Arabidopsis*. The Phylogenetic tree was constructed with iTOL software by using the neighbor-joining method.

For searching the AP2/ERF family genes in wheat genome, we used the HMM (Hidden Markov Model) search which uses HMM profiles with the AP2 domain of *Arabidopsis* (Pfam ID: PF00847) as a query to search for wheat AP2/ERF gene in the overall plant database. Firstly, we identified a total of 380 genes with potential coding AP2/ERF domains in the wheat genome. To purify our search, we used SMART program search to identify the exact genes with AP2/ERF domains and obtained 322 genes with AP2/ERF domains. For further confirmation, we used CDD^3^ model to confirm all genes with AP2/ERF domains listed in Supplementary Table 2. Almost all the genes have at least one AP2 domain. According to the bias of the Neighbor-Joining (NJ) phylogenetic tree (Figure 1), we divided the genes into 12 subfamilies namely Ia-IVc. Subfamily-Ia have 9.32%, subfamily-Ib 13.6%, subfamily-Ic 7.14%, subfamily-IIa 9.63%, subfamily-IIb 9.94%, subfamily-IIc 4.66%, subfamily-IIIa 4.35%, subfamily-IIIb 8.0%, subfamily-IIIc 11.8%, subfamily-IVa 4.03%, and other two subfamilies namely IVb 8.70% and IVc have 8.38% genes, respectively. Through searching in different databases, it was found that the gene members of subfamily-Ia and subfamily-IIc were more responsive to different abiotic stresses so we randomly selected some genes of these two subfamilies and performed further analysis. We used the online ExPASy server to calculate the length, molecular weight, and isoelectric point of these proteins.

The protein length ranged from 167 to 461 amino acids, and the molecular weight ranged from 18336.2 to 49741.7 kDa. Similarly, as shown in Supplementary Table 3, the isoelectric point (pI) also ranged from 4.47 to 11.69.

### Phylogenetic Analysis of Wheat AP2/ERF Gene Family

In order to analyze the evolutionary relationship and classification of AP2/ERF genes of wheat with *Arabidopsis* and Rice AP2/ERF, we performed the phylogenetic analysis and constructed the unrooted tree using MEGA 5.0 with the Neighbor-joining method. The outcomes of the phylogenetic tree showed 12 different clades (designated as Ia-IVc) (Figure 1). From subfamily-Ia to subfamily-IIa were grouped under the DREB subfamily with 30, 44, 23, and 31 members, respectively, and from subfamily-IIb to subfamily-IVa were grouped under the ERF subfamily with 32, 15, 14, 26, 38, and 13 members, respectively, as in *Arabidopsis* and rice (Nakano et al., 2006). The bootstrap values for all subfamilies were not very high as reported in previous research on *Arabidopsis* ERF proteins (Nakano et al., 2006). To verify the NJ-phylogenetic tree results, we also drawn another phylogenetic tree by using Maximum Parsimony (MP) analysis for each subfamily and found that all AP2/ERF members were placed in the same subfamilies (Supplementary Figure 1).

### Chromosomal Location, Synteny, and Collinearity Relationship Analysis Between Wheat, Rice, and *Arabidopsis* of AP2/ERF Gene Family

**FIGURE 2:**
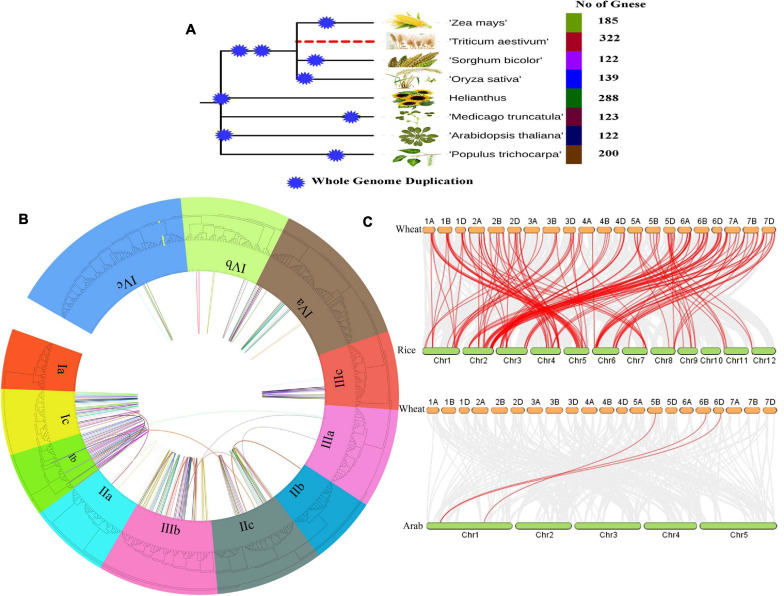
**(A)** Total number of AP2/ERF genes in different crops during Whole Genome Duplication analysis, **(B)** The Phylogenetic tree and Microsynteny analyses of AP2/ERF genes family among wheat, rice, *and Arabidopsis*. **(C)** Collinearity relationship of AP2/ERF genes in wheat, rice, and *Arabidopsis*.

The position of the AP2/ERF genes on the chromosome and the syntenic analysis between A, B, and D subgenomes are shown in Supplementary Figure 2. As shown in the figure, all the TaAP2/ERF genes are distributed irregularly on all 21 chromosomes. The total number of genes present on each chromosome varies from as low as 10 (on Chromosome 5B) to as high as 22 (on Chromosome 6B). Of all the 322 genes, 320 genes were located on all chromosomes and 2 genes were located on the Scaffold. Among the 320 genes on chromosomes, the percentage of genes in subgenomic D was the highest, at 109 (34.06%), genes in subgenomic B were 108 (33.75%) and genes in subgenomic A were 103 (32.19%). As for chromosomes, most of the chromosomes had 3–5% genes and only four chromosomes (1B, 1D, 6A, 6B) had more than 6% genes. It was found that most of the AP2/ERF genes from subfamily-Ia to subfamily-IVc were located at the telomeres ends of most chromosomes such as 1A, 1B, 1D, 2A, 2B, 2D, 4A, 4B, 4D, 6A, 6B, and 6D and many other genes were unevenly distributed on all 21 chromosomes as shown in Supplementary Figure 2. All homologous genes of AP2/ERF on 21 chromosomes were also analyzed and demonstrated in the middle circle of the Figure 2B. All AP2/ERF homologs were also entangled with chromosomal translocation and the pericentromeric inversion processes described and their crosslinking is shown in the inner circle of Figure 2B.

To analyze the orthologous relationships and evolutionary origin of TaAP2/ERF genes of wheat with rice and *Arabidopsis* AP2/ERF, the molecular history of AP2/ERF family members were further analyzed. A tightly conserved collinearity relationship was found between the regions among the wheat, rice, and *Arabidopsis* (Figure 2C). The collinearity analysis between the wheat, rice and *Arabidopsis* showed a total of 117 orthologous pairs of AP2/ERF from 257 genes (Figure 2C and Supplementary Table 4). A total of 118 genes of wheat, 136 gene of rice and 3 genes of *Arabidopsis* were present in these orthologous pairs. These collinearity results showed that there is a close similarity between the syntenic orthologous groups and the phylogenetic relationship (Figures 2A–C).

### *Cis*-Acting Elements Analysis of AP2/ERF Gene Family

**FIGURE 3:**
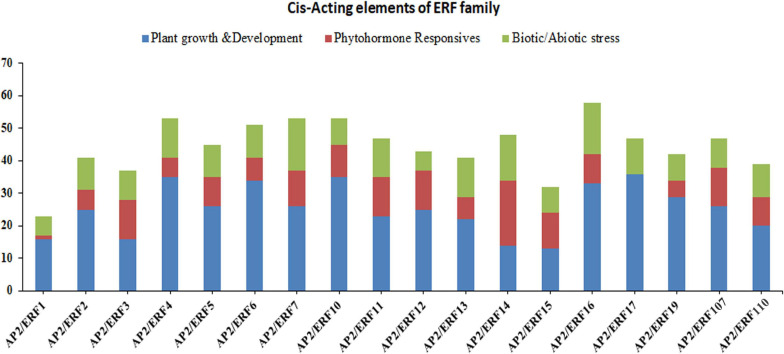
Investigation of *cis*-acting element numbers of TaAP2/ERF genes in Wheat. *Cis*-acting elements of each category represented by different colors.

All plants have two important regulatory mechanisms; *cis*-acting elements and *trans*-acting elements. Both of these mechanisms interact with each other to increase or decrease the expression of the genes. The *cis*-acting elements may be present in both coding and non-coding sequence of the gene. The *cis*-acting elements in the promoter region play a vital role in plant regulation control, stress-responsive gene expression patterns, tissue-specific gene expression, and also play a role in different stimulus-responsive genes. In order to fully understand the potential role of AP2/ERF genes in subfamily-Ia and subfamily-IIc, we downloaded the 2000 bp upstream sequence from the start codon (ATG) of the genomic DNA sequence and analyzed it in the PlantCARE database. The results showed that different types of *cis*-acting elements were found in different genes (Figure 3 and Supplementary Table 5). The presence of different numbers and types of *cis*-acting elements in AP2/ERF genes recommends that these genes may be involved in different regulatory mechanisms. We divided these *cis*-acting elements into three different categories, i.e., biotic/abiotic stress responses, phytohormones responses and growth and developmental responses (Figure 3). Under the growth and development, a different type of *cis*-acting elements was found; for instance, CAT-box involved in meristem expression, Box-4 and MRE involved in light responsiveness, and O2-site involved in zein metabolism regulation. The results showed that in growth and development category, CAT-box dominates by 93.5% followed by O2-site (2.69%), Box-4 (2.26%) and MRE (1.55%), respectively. In the phytohormone responsive category, TCA-Element involved in salicylic acid responses, TGACG-motif and CGTCA-motif in MejA responses, ABRE in ABA responses, ERE in ethylene responses and GARE-motif in gibberellin responses. It was found that ABRE class was the highest (40.16%), followed by TGACG-motif (23.69%), CGTCA-motif (22.09%), ERE (6.02%), TCA-Elements (5.62%) and GARE-motif (2.41%). Category for biotic/abiotic stresses also contained a different type of *cis*-acting elements such as MBS involved in drought responses, TC-rich repeats, MYB and MYC involved in defense and stress responses, As-1 in root-specific expression, ARE in anaerobic induction responses and LTR in low-temperature responses. In this category, MYB contained a major portion (30.29%) followed by MYC (29.64%), As-1 (19.22%), ARE (8.14%), LTR (5.54%), MBS (4.88%), and TC-rich repeats have (2.28%). Taken together, the genes of subfamily-Ia and IIc of AP2/ERF family may have a potential role in biotic and abiotic stress control (Figure 3).

### Gene Structure and Conserved Motif Analysis of AP2/ERF Gene Family

**FIGURE 4:**
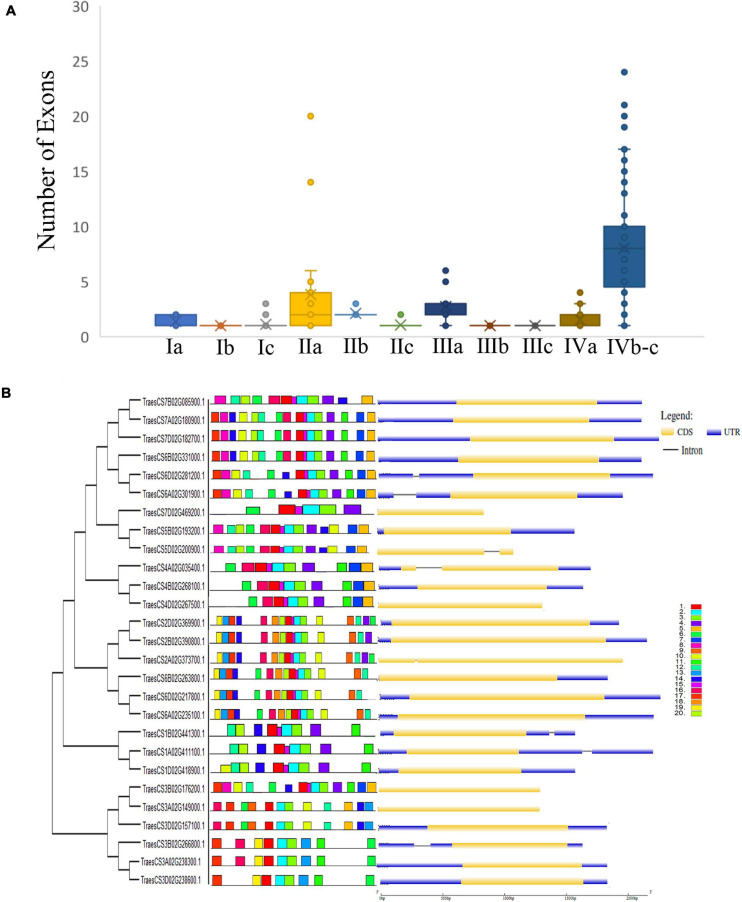
Distribution of numbers of exons, conserved motifs and Gene structure and of TaAP2/ERF genes in Wheat. **(A)** Presence of different numbers of Exons in different groups of TaAP2/ERF gene family in Wheat. **(B)** Phylogenetic relationship, conserved motifs located on each gene with the relative combined *p*-value and gene structure of Wheat TaAP2/ERF genes.

To understand the complete nature of the genes and their structure, gene structure analysis, or intron/exon analysis was performed, as the presence and position of the intron and exon in the genes could be used to understand the phylogenetic relationship between the genes (Fedorov et al., 2002; Babenko et al., 2004). For gene structure analysis, we used CDS sequence and genomic sequence of the corresponding gene and analyzed it in the GSDS online tool. As shown in a previous study, most *Arabidopsis* ERF genes have no introns (Sakuma et al., 2002), which strongly corroborates the findings of our study (Figures 4A,B). Most genes in the two subfamilies had fewer introns while other members such as the genes TaAP2/ERF5, TaAP2/ERF6, TaAP2/ERF9, TaAP2/ERF10, TaAP2/ERF15, TaAP2/ERF19, TaAP2/ERF20, and TaAP2/ERF110 contained one intron (Figure 4B). Genes without intron or one intron showed similar functions and have similar evolutionary processes. These results indicate that these genes have remained conserved during evolutionary processes and have a high degree of functional similarities.

For the conservative motif analysis of AP2/ERF genes of subfamily-I and IIc, we used the protein sequence of the genes and analyzed it with the MEME online tool. We analyzed 20 motifs in each gene and named them from motif 1 to motif 20 (Figure 4B). As shown in Figure 4, the genes were divided into different groups ranging from 1 to 12. The genes in groups 3, 5, and 10 contain 14 motifs, the group 2 and 7 contain 16 motifs, group 1 and 11 contain 12 motifs, group 9 and 12 contain 9 motifs, while the groups 4, 6, and 8 contain 6, 10, and 15 motifs, respectively. Group 7 and 8 contain several motifs but on the other side, group 4 only contains 6 motifs. Genes in these groups contain the same motifs which suggests that they may have the same function. It was also observed from the results that some motifs were conserved in their groups whereas, some motifs such as motif1, motif2, motif3, motif10, and motif18 were distributed in all genes. The presence of these motifs in all genes shows that these motifs may be necessary for their basic functions. Similar motifs and similar intron/exon structures among the genes revealed that these genes may have the same functions.

### GO Annotation Analysis

**FIGURE 5:**
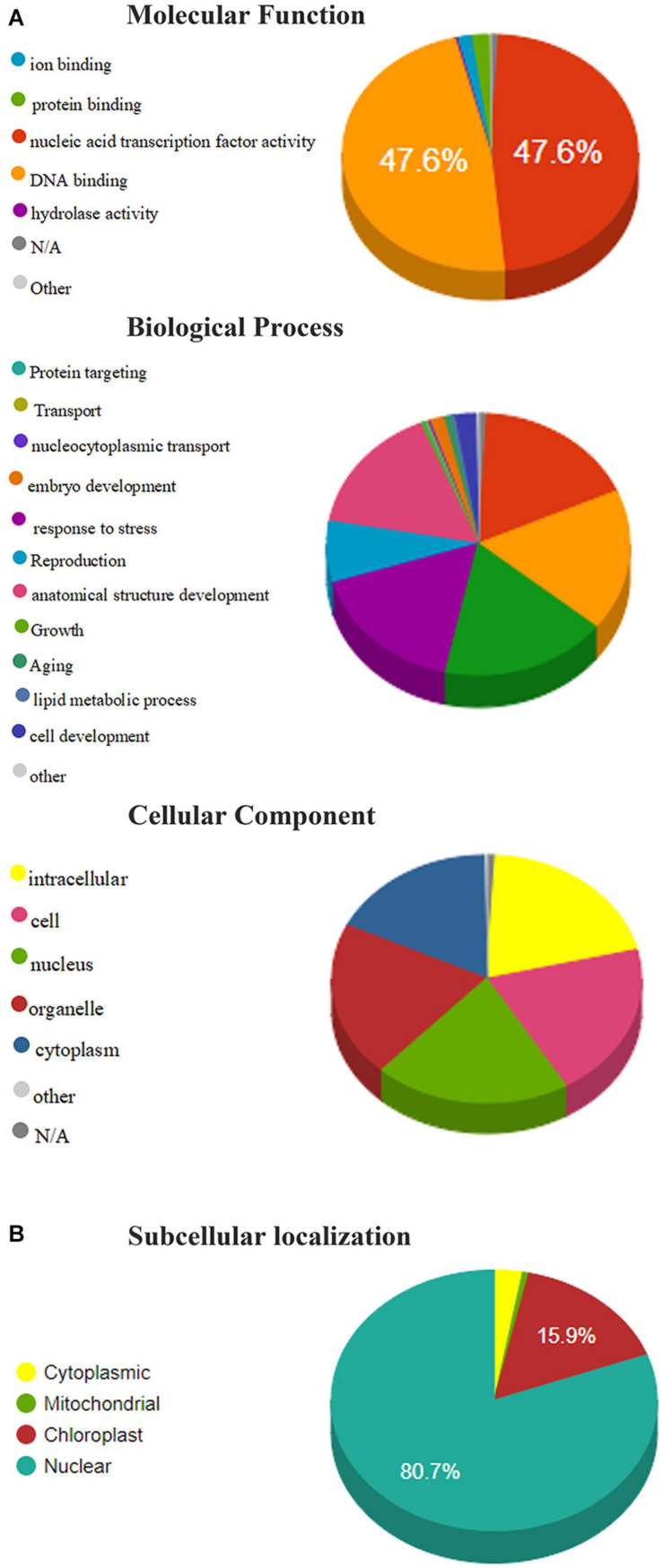
**(A)** Molecular functions, biological processes, and cellular component of members of the TaAP2/ERF family based on gene ontology (GO) analysis. **(B)** Subcellular localization of TaAP2/ERF protein in Wheat.

Gene ontology annotation analysis is used to predict the functions and subcellular localization of putative TaAP2/ERF protein in wheat. 322 TaAP2/ERF proteins were grouped into 36 functional groups based on amino acid similarities and categorized into three main ontologies, namely Molecular functions, Biological process, and cellular component (Supplementary Table 6). In the molecular functions annotations, we analyzed that more than 95% of annotated proteins predicted their functions in the nucleic-acid binding activity, followed by protein-binding (1.92%) and ion binding (1.58%) (Figure 5A). In the biological process annotation, TaAP2/ERF protein percentage annotated with the biosynthetic process (17.54%), cellular nitrogen compound metabolic process (17.54%) followed by signal transduction (17.44%), response to stress (16.74%), anatomical structure development (15.90%), and reproduction (7.58%). Subsequently, predicted TaAP2/ERF proteins are also annotated with cell differentiation (2.30%), embryo development (1.52%), and aging (>1%) in biological process annotation (Figure 5A). The cellular component annotations showed that the TaAP2/ERF proteins annotated with the nucleus, intracellular, cell, and organelles having the same percentage (20.29%) and cytoplasm (17.57%) (Figure 5A).

Furthermore, our results showed that most of the TaAP2/ERF proteins were localized in the nucleus, chloroplast, cytoplasm, and mitochondria (80.7%, 15.9%, 2.8%, 0.62%, respectively) (Figure 5B).

### Expression Profile of TaAP2/ERF Genes Under Abiotic Stresses

**FIGURE 6:**
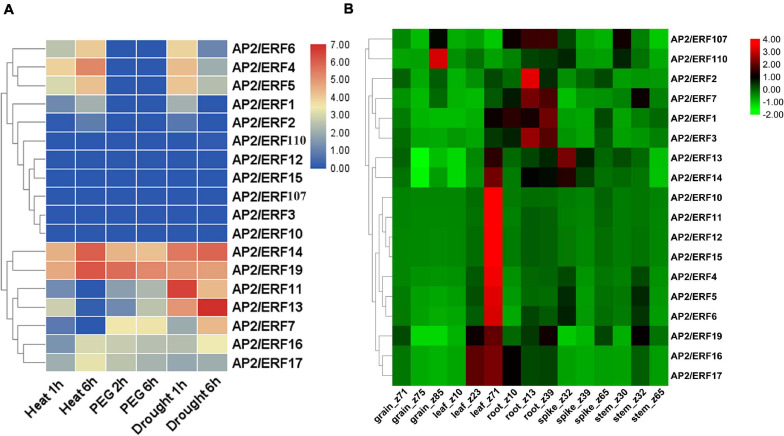
Heat map of TaAP2/ERF genes in Wheat under different abiotic stresses and various tissues at different developmental stages. **(A)** The TPMs were calculated for expression values from RNA-seq data. Different colors represent expression level; The red, violet, and blue color indicated high expression, low expression, and no expression, respectively. **(B)** Log_2_ transformed (FPKM + 1) expression values were used to create the heat map. The highest level of expression is represented by red, while the low level is represented by green.

The function of genes often has a great relationship with the expression pattern of genes. By acquiring the RNA-seq expression data from the expVIP database and WheatExp database, we studied the expression pattern of 18 AP2/ERF genes of wheat in various tissues (leaf, stem, spike, and grain) and under different abiotic stresses (Figures 6A,B and Supplementary Table 7). According to the RNA-seq data, 4 genes including TaAP2/ERF14, TaAP2/ERF16, TaAP2/ERF17, and TaAP2/ERF19 exhibited their expression under all stresses, 8 genes including TaAP2/ERF1, TaAP2/ERF2, TaAP2/ERF4, TaAP2/ERF5, TaAP2/ERF6, TaAP2/ERF7, TaAP2/ERF9, and TaAP2/ERF11 were expressed under most of the stresses while 6 genes TaAP2/ERF3, TaAP2/ERF10, TaAP2/ERF12, TaAP2/ERF15, TaAP2/ERF107, and TaAP2/ERF110 did not show any expression under the tested stresses (Figure 6A). Furthermore, Figure 6B showed that all the tested 18 TaAP2/ERF genes were expressed in all tissues at different stages. Except for TaAP2/ERF107 and TaAP2/ERF110, all other genes showed higher expression in leaf tissues. TaAP2/ERF1, TaAP2/ERF2, TaAP2/ERF3, TaAP2/ERF7, TaAP2/ERF13, TaAP2/ERF14, TaAP2/ERF19, and TaAP2/ERF107 were expressed in root tissues. 6 genes TaAP2/ERF4, TaAP2/ERF5, TaAP2/ERF6, TaAP2/ERF13, TaAP2/ERF14, and TaAP2/ERF110 expressed in spike tissues and 5 genes TaAP2/ERF5, TaAP2/ERF7, TaAP2/ERF9, TaAP2/ERF107 and, TaAP2/ERF110 were expressed in the stem tissues. Only two genes TaAP2/ERF107 and, TaAP2/ERF110 were expressed in grain tissues.

### Expression Pattern Analysis of Subfamily Ia and IIc Genes Under Heat, Salt, and Drought Stresses

**FIGURE 7:**
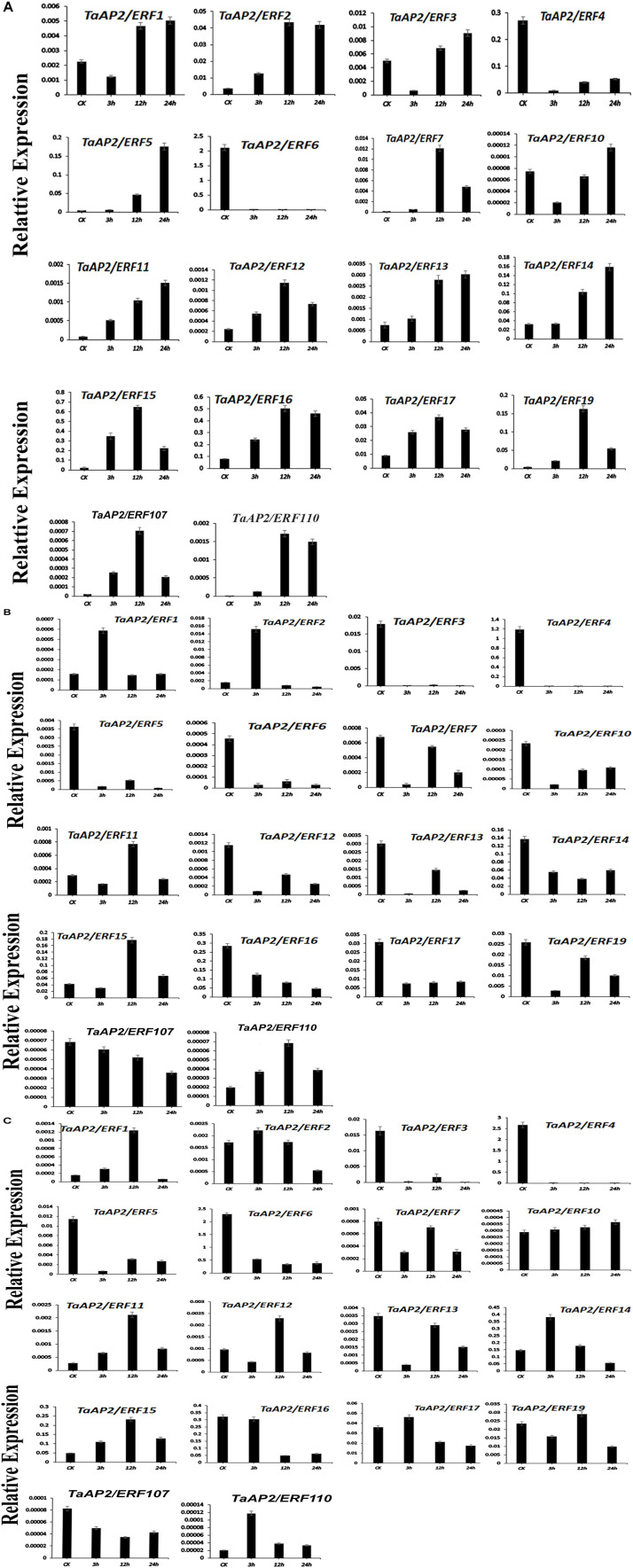
The relative expression level of TaAP2/ERF genes under different Stresses **(A)** Under Heat Stress **(B)** Under NaCl Stress **(C)** Under Drought Stress. Error bars indicate the standard error (SE) between three replicates.

To confirm the analysis of the expression patterns of genes in subfamily Ia and IIc, 18 different genes were randomly selected and their expression was tested by qRT-PCR under heat, salt and drought stress treatments (Figures 7A–C). The results showed that under heat treatment, most of the genes exhibit a higher expression level than control. Besides, the expression level of TaAP2/ERF1, TaAP2/ERF3, TaAP2/ERF5, TaAP2/ERF10, TaAP2/ERF11, TaAP2/ERF13, and TaAP2/ERF14 were increasing from CK to 24 h. However, the expression level of TaAP2/ERF2, TaAP2/ERF12, TaAP2/ERF17, TaAP2/ERF7, TaAP2/ERF19, TaAP2/ERF15, TaAP2/ERF16, TaAP2/ERF107, and TaAP2/ERF110 increased continuously from CK to 12 h and later decreased at 24 h. Both TaAP2/ERF4 and TaAP2/ERF6 genes did not show any significant expression under the heat treatment (Figure 7A).

Under Salt stress, most genes such as TaAP2/ERF3, TaAP2/ERF4, TaAP2/ERF5, TaAP2/ERF6, TaAP2/ERF10, TaAP2/ERF12, TaAP2/ERF13, TaAP2/ERF14, TaAP2/ERF17, TaAP2/ERF7, TaAP2/ERF19, TaAP2/ERF16, and TaAP2/ERF107 did not show higher expression levels. Two genes TaAP2/ERF1 and TaAP2/ERF2, showed a higher expression level at 3 h when compared to CK and later decreased their expression at 12 and 24 h. The expression levels of TaAP2/ERF11, TaAP2/ERF15, and TaAP2/ERF110 gradually increased at 12 h and then decreased at 24 h (Figure 7B).

Most of the genes are expressed randomly under drought stress. We divided them into three groups; in the 1st group of genes, TaAP2/ERF2, TaAP2/ERF14, TaAP2/ERF17, and TaAP2/ERF110 showed a similar higher expression pattern at 3 h and then their expression was decreased at 12 and 24 h. In the 2nd group of genes, TaAP2/ERF1, TaAP2/ERF11, TaAP2/ERF12, TaAP2/ERF15, and TaAP2/ERF19, continuously higher expression levels at CK, 3 and 12 h was observed and, after that the expression level decreased at 24 h (Figure 7C). The expression level of TaAP2/ERF10 increased immediately at 3 h under drought stress and continued to increase at 12 and 24 h. Having said that, eight genes namely TaAP2/ERF3, TaAP2/ERF4, TaAP2/ERF5, TaAP2/ERF6, TaAP2/ERF13, TaAP2/ERF7, TaAP2/ERF16, and TaAP2/ERF107 did not show any remarkable expression levels under drought stress conditions.

## Discussion

In plants, AP2/ERF superfamily has an extensive range of transcription factors members, which plays a vital role in the regulation of transcriptional processes related to biotic, abiotic stresses, and developmental processes, which also interferes with seed germination, fruit ripening, and development of flower and leaf senescence. These transcription factors also respond to pathogen invasion, high and low temperature, drought and salt stresses (Klucher et al., 1996; Wang et al., 2007, 2008; Hattori et al., 2009; Woo et al., 2010; Yang et al., 2011; Schmidt et al., 2013; Zhu et al., 2014). As AP2/ERF transcription factors mainly exist in plants (Rashid et al., 2012; Licausi et al., 2013), the study of these transcription factors can provide us with insights into the function and evolution of these transcription factors in different plant species. With technological development, more and more plant genome data from different plant species have been released and in-depth research has been conducted on the AP2/ERF superfamily. So far, the AP2/ERF superfamily in *Arabidopsis* (Nakano et al., 2006), rice (Rashid et al., 2012), wheat (Zhao et al., 2019), *Triticum durum* (Faraji et al., 2020), foxtail millet (Lata et al., 2014), Chinese cabbage (Song et al., 2013), cabbage (Thamilarasan et al., 2014), *Brachypodium distachyon* (Chen et al., 2016), *Musa balbisiana*, *Musa acuminate* (Lakhwani et al., 2016), Switchgrass (Wuddineh et al., 2015), castor bean (Xu et al., 2013), peach (Zhang et al., 2012), poplar (Zhuang et al., 2008), and grapevine (Licausi et al., 2010) have successfully been analyzed. However, there is not enough research has been conducted on TaAP2/ERF family. In the current study, we accomplished a comprehensive analysis of the hexaploid wheat TaAP2/ERF gene family together with genome-wide identification, gene structure, gene locations, *cis*-acting elements analysis, motif analysis, gene ontology, synteny relationships and expression pattern analysis under various stress conditions. A total of 322 TaAP2/ERF genes were identified in wheat, among which, there is at least one conserved AP2/ERF domain (Supplementary Table 2). Exceptionally, the total TaAP2/ERF genes in wheat exceed that of *Arabidopsis* (147 genes), rice (164 genes), foxtail millet (171 genes), *T. durum* (271 genes) and maize (210 genes) (Nakano et al., 2006; Liu et al., 2013; Lata et al., 2014; Faraji et al., 2020), which strongly strengthened the hypothesis that in plants ploidy level could increase the genome size. The number of gene members in almost all subfamilies is mostly higher than that of *Arabidopsis*, rice, maize and foxtail millet. Recent studies have shown that in plants, segmental duplication and tandem duplication events had a contribution to the enlargement of AP2/ERF family, suggesting that stress is the central force affecting the evolution of AP2/ERF family (Nakano et al., 2006; Liu et al., 2013; Lata et al., 2014). The result of collinearity analysis shows that there is a close similarity between the syntenic orthologous groups and the phylogenetic relationship (Figures 2A–C).

The presence of conserved motifs in gene transcription factors has a vital role in gene function (Sakuma et al., 2002). The motifs are also involved in transcriptional activity, DNA binding, and protein interaction (Liu et al., 1999). It is already investigated that a motif, ERF-associated amphiphilic repression (EAR) having a repression domain in the C-terminal regions of the repressor–type ERF proteins play a critical role in different biological functions by implying a negative role on genes that are involved in stress, developmental and hormonal signaling pathways (Fujimoto et al., 2000; Ohta et al., 2001). In *Arabidopsis*, fifty motifs were detected outside the AP2/ERF domain (Nakano et al., 2006). In our study, we identify twenty motifs in TaAP2/ERF proteins (Figure 4) that are similar to be presented in *T. durum* (Faraji et al., 2020), *Arabidopsis* (Sakuma et al., 2006), *B. distachyon* (Cui et al., 2016). The genes within the groups contain the same motifs, so it can be suggested that they may have the same functions. The results also revealed that some motifs were conserved in their groups whereas, some motif such as motif1, motif2, motif3, motif10, and motif18 were distributed among all the genes. The analysis of gene structure gives us more insight into the evolution and functions of genes. In this study, most genes in the same group have the same position of the exon-intron structure, but there are some irregularities, which may be due to loss, gain, or sliding of introns during the formation of AP2/ERF gene family (Rogozin et al., 2005). In the present study, the gene TaAP2/ERF5, TaAP2/ERF6, TaAP2/ERF9, TaAP2/ERF10, TaAP2/ERF15, TaAP2/ERF19, TaAP2/ERF20 and TaAP2/ERF110 have only 1 intron Figure 4B, suggesting the intron loss in evolution process. The previous studies showed that in plants during evolution, selection pressure is the cause of intron loss or gain, and genes evolve themselves into multiple exon-intron structures to perform typical functions (Mattick, 1994; Wang et al., 2016). The lack of introns in genes would speed up the evolution process by gene copying (Lecharny et al., 2003). The genes having no intron or one intron showed to be similar in their function and shared a similar evolution process. These results showed that these genes remain conserved under the evolution process and possess highly functional similarities. The presence of a similar motif and similar intron/exon structure between the genes revealed that these genes might have the same functions.

Plants have two important regulatory mechanisms; *cis*-acting elements and *trans*-acting elements. Both of these mechanisms interact with each other to increase or decrease the expression of a given gene. The *cis*-acting elements may be present in both coding and non-coding sequence of the gene. The *cis*-acting elements present in the promoter region play a vital role in plant regulation control, having involvement in the stress-responsive gene expression patterns, tissue-specific gene expression, and also have a role in different stimulus responsive genes. The presence of different numbers and types of *cis*-acting elements in AP2/ERF genes specify the different regulatory mechanisms in which these genes may be involved. It has been largely supposed that under stress conditions AP2/ERF family protein can bind to GCC-box with their MG metal ion in the AP2 domain to alter the expression of the targeted genes (Shigyo et al., 2006; Lata et al., 2014; Mathur et al., 2020). The DREB proteins encoding genes are significantly responsive to drought and cold, so overexpression of these genes in plants could increase salt, drought, and cold tolerance (Shigyo et al., 2006; Lata et al., 2014; Shu et al., 2016). Additionally, DRE is the central sequence of those genes that are involved in cold and drought stress-responsive (Lata and Prasad, 2011; Heidari, 2019; Xu et al., 2020). In this study, we identified different types of *cis*-acting elements like growth and development, CAT-box involved in meristem expression, Box-4, and MRE play role in light responsiveness, and O2-site essentially functions in zein metabolism regulation. The *cis*-elements for phytohormone responses are TCA-Element participate in salicylic acid responses, TGACG-motif and CGTCA-motif have a role in MejA responses, ABRE participate in ABA responses, ERE involves in ethylene responses and GARE-motif involve in gibberellin responses. For biotic/abiotic stresses there are also different type of *cis*-acting elements like MBS involved in drought responses (Manzoor et al., 2020), TC-rich repeats, MYB and MYC functions in defense and stress responses, As-1 involved in root specific expression, ARE involved in anaerobic induction responses and LTR has a role in low-temperature responses Figure 3. Several tested ERF genes were upregulated in cassava under osmotic and salt stresses (Fan et al., 2016). AP2/ERF has a known vital role in plant growth regulation, development and coping with different environmental stresses and also, has a role in different signal transduction pathways (Mizoi et al., 2012).

The Expression pattern of genes had a great correlation with its functions. The tissue-specific expression data at a given developmental stage is important for identifying the gene functions in which they are involved. In our study, the expression pattern of different genes under multiple stresses and in various wheat tissues was investigated to understand the potential role of these genes during stress and development response. Most of the genes under study showed their expression in different developmental tissues indicating their role in these tissues (Figures 6A,B). However, the functions of TaAP2/ERF is not still completely known. Therefore, to completely understand the functions of TaAP2/ERF family under different environmental stresses, we carried out the qRT-PCR of 18 different genes under different biotic/abiotic stresses. Most of the genes are expressed under these different stresses but their expression pattern are different under different stresses same as in *T. durum* which strengthened our results (Figures 7A–C; Faraji et al., 2020). TaAP2/ERF1, TaAP2/ERF3, TaAP2/ERF5, TaAP2/ERF10, TaAP2/ERF11, TaAP2/ERF13, and TaAP2/ERF14 show rapid response and their expression continuously increased under heat stress. On the other hand, TaAP2/ERF2, TaAP2/ERF12, TaAP2/ERF17, TaAP2/ERF7, TaAP2/ERF19, TaAP2/ERF15, TaAP2/ERF16, TaAP2/ERF107, and TaAP2/ERF110 also shows quick response under stress at 3 and 12 h but at 24 h shows no significant expressions. Under Salt stress, most of the genes show no significant expression. TaAP2/ERF11, TaAP2/ERF15 and TaAP2/ERF110 show higher expression levels than all other genes, and TaAP2/ERF1 and TaAP2/ERF2 also show a quick response under salt stress at 3 h but on prolonged stress theses gene also did not show any significant expression. TaAP2/ERF2, TaAP2/ERF14, TaAP2/ERF17, and TaAP2/ERF110 show a quick response at 3 h under drought stress and then their expression starts declining at prolonged stress. Five genes TaAP2/ERF1, TaAP2/ERF11, TaAP2/ERF12, TaAP2/ERF15, and TaAP2/ERF19 show the expression at 3 and 12 h but at 24 h the expression of these genes also decreased. The expression level of TaAP2/ERF10 immediately increased under drought stress at 3 h and continuously increased at 12 and 24 h Figure 7C. TaAP2/ERF gene family is supposed to have a critical role in signal transduction and transcriptional adjustment because most of the genes of this family are predicted to be present in the nucleus as compared with *T. durum* which is a close relative to *T. aestivum* Figure 5B (Faraji et al., 2020). As it is clear from the previous results that AP2/ERF gene family plays a critical role in plant growth and development (Scheres and Krizek, 2018; Dipp-álvarez and Cruz-Ramírez, 2019; Jiang et al., 2019; Wang et al., 2019). So, our study about AP2/ERF genes in wheat will be useful for improving the wheat crop from different biotic and abiotic stresses in the future.

## Conclusion

Current finding on the latest genome sequence of wheat (*Triticum aestivum* L.) provides plant biologists the new knowledge of functional genomics. In this study, genome-wide analysis has been conducted and a total of 322 putative genes of TaAP2/ERF were compared with 147 *Arabidopsis* and 164 rice AP2/ERF genes. The 322 TaAP2/ERF genes were grouped into 12 subfamilies, Ia-IVc as compared with previous studies (Nakano et al., 2006). We performed the phylogenetic, *cis*-acting elements, conserved motif, intron-exon analysis as well as chromosomal location, Gene ontology (Molecular functions, Biological process, and cellular component), microsynteny, collinearity, promoter region and qRT-PCR analysis of eighteen genes undergoing different environmental conditions. Genome distribution and chromosomal localization indicate that the expansion of TaAP2/ERF might be contributed by tandem duplication. Our finding provides us a strong base for evolutionary history, and molecular characterization of AP2/ERF genes in wheat, especially under different stress conditions.

## Data Availability Statement

All datasets generated for this study are included in the article/Supplementary Material.

## Author Contributions

MWR conceived and designed the experiments. LS, LY, CC, XDM, LX, MAM, MA, and SR contributed to reagents, materials, and analysis tools. HS and CM guided the whole manuscript. MWR and JL wrote the article. All authors read and approved the final manuscript.

## Conflict of Interest

The authors declare that the research was conducted in the absence of any commercial or financial relationships that could be construed as a potential conflict of interest.

## Footnotes

1 http://www.wheatgenome.org

2 https://plants.ensembl.org/index.html

3 http://pfam.xfam.org

4 http://smart.embl-heidelberg.de

5 http://expasy.org/

6 http://rice.plantbiology.msu.edu/

7 http://www.arabidopsis.org

8 http://gsds.cbi.pku.edu.cn/

9 http://meme-suite.org/tools/meme

10 http://bioinformatics.psb.ugent.be/webtools/plantcare/html

11 http://www.wheat-expression.com

12 http://wheat.pw.usda.gov/wheatExp

## Supplementary Material

Supplementary Figure 1 Maximum Parsimony (MP) analysis of TaAP2/ERF gene family in wheat.

Supplementary Figure 2 Chromosomal distribution of TaAP2/ERF family genes.

Supplementary Table 1 List of primers used in qRT-PCR analysis.

Supplementary Table 2 List of all putative genes having AP2 domain.

Supplementary Table 3 Characteristics features of TaAP2/ERF genes of subfamily-Ia and IIc.

Supplementary Table 4 List of TaAP2/ERF orthologous pair identified in *Arabidopsis* and rice.

Supplementary Table 5 List of *Cis*-acting elements of TaAP2/ERF genes of subfamily-Ia and IIc.

Supplementary Table 6 Molecular function, biological process, cellular components and subcellular localization of TaAP2/ERF gene family in wheat.

Supplementary Table 7 RNA-seq data acquired from WheatExp database.

## Abbreviations

AP2/ERF: APETALA2/Ethylene Responsive Factor
AP2: APETALA2
DREB: dehydration-responsive element-binding
ERF: ethylene response factors
ET: ethylene
SA: salicylic acid
ABA: abscisic acid
NaCl: sodium chloride
HMM: Hidden Markov Model
GO: gene ontology
PEG: polyethylene glycol
TF: transcription factors
MEME: Multiple Em for Motif Elicitation
NJ: neighbor-joining
MW: molecular weight
pI: isoelectric point
GSDS: gene structure display server
MCSscanX: multiple collinearity scan toolkit
TPM: transcripts per million
RNA: ribonucleic acid
MP: maximum parsimony
qRT-PCR: real-time quantitative reverse transcription PCR
gDNA: genomic DNA
CDS: coding sequence.
