# Supplementary figures and images for: Expansion and Molecular Characterization of AP2/ERF Gene Family in Wheat (Triticum aestivum L.)
Source: Front Genet. 2021 Mar 31;12:632155. doi: 10.3389/fgene.2021.632155 (PMC8044323; doi:10.3389/fgene.2021.632155)

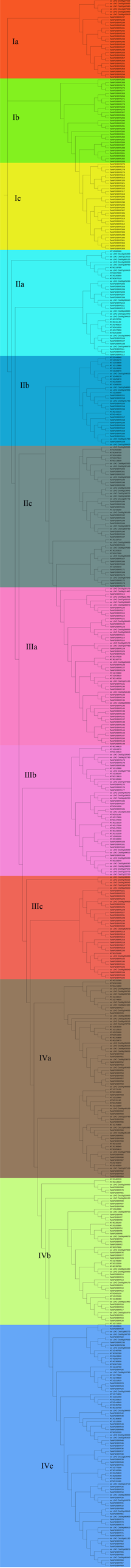

Supplement: Supplementary Figure 1 — Maximum Parsimony (MP) analysis of TaAP2/ERF gene family in wheat. [file Image_1.TIF]

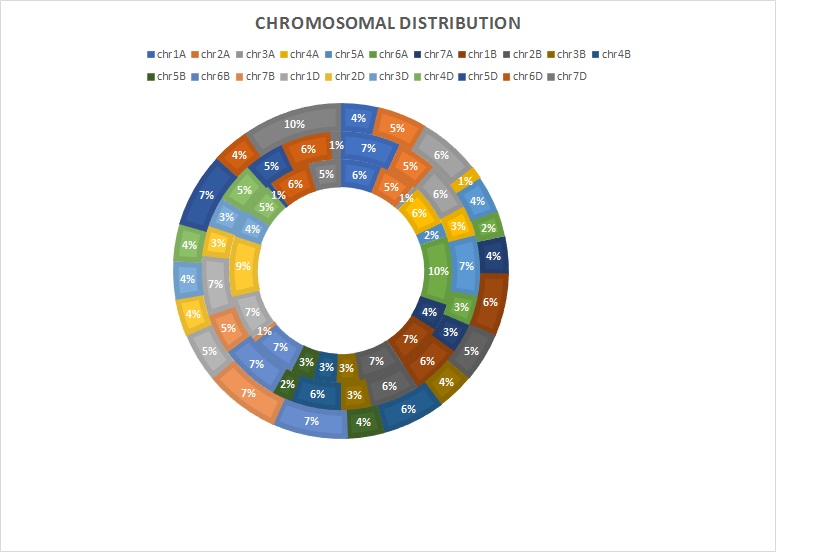

Supplement: Supplementary Figure 2 — Chromosomal distribution of TaAP2/ERF family genes. [file Image_2.JPEG]
